# Preparation of Furfural From Xylose Catalyzed by Diimidazole Hexafluorophosphate in Microwave

**DOI:** 10.3389/fchem.2021.727382

**Published:** 2021-09-01

**Authors:** Ting Huang, Kun Yuan, Xu-Liang Nie, Jing Chen, Huang-Xian Zhang, Jin-Zhu Chen, Wan-Ming Xiong

**Affiliations:** ^1^ College of Science, Jiangxi Agricultural University, Nanchang, China; ^2^ Knowledge Innovation Team of Organic Functional Materials and Agricultural Applications of Nanchang City, Jiangxi Agricultural University, Nanchang, China; ^3^ School of Information and Engineering, Jiangxi Agricultural University, Nanchang, China

**Keywords:** xylose, furfural, diimidazole hexafluorophosphate, camphorsulfonic acid, microwave

## Abstract

In this work, functionalized alkyl imidazolium hexafluorophosphate ILs were synthesized and characterized; then, they were applied in the conversion of xylose to furfural under the microwave method. The results showed that when C_n_MF was used as a catalyst, an acidic environment was provided to promote the formation of furfural. In addition, the heating method, the solvent, and the different structures of cations in the ionic liquid influenced their catalytic activity. In an aqueous solution, the yield of furfural obtained using the microwave method was better than that of the conventional heating method, and the catalytic activity of diimidazole hexafluorophosphate was better than that of monoimidazole. Meanwhile, for the diimidazole hexafluorophosphate, the change of the carbon chain length between the imidazole rings also slightly influenced the yield. Finally, the optimal yield of 49.76% was obtained at 205°C for 8 min using 3,3′-methylenebis(1-methyl-1H-imidazol-3-ium), C_1_MF, as a catalyst. Mechanistic studies suggested that the catalytic activity of C_1_MF was mainly due to the combined effect of POF_n_ (OH)_3-n_ and imidazole ring. Without a doubt, the catalytic activity of C_1_MF was still available after five cycles, which not only showed its excellent catalytic activity in catalyzing the xylose to prepare the biomass platform compound furfural but also could promote the application of functionalized ionic liquids.

## Introduction

As one of the recognized renewable energy sources in the world, bioenergy has the characteristics of green, low carbon and low cost. Therefore, in the context of today’s energy shortage, how to realize the efficient development and utilization of renewable bioresources has attracted extensive attention from researchers. Furfural, also known as furaldehyde, is an important biomass platform compound. Its derivatives are widely used in many industries, making it one of the most promising biomass-derived platforms (Chi et al., 2009; Sui et al., 2018; Chen et al., 2021; Krzelj et al., 2021).

Several years ago, the preparation of furfural still relied on traditional acid-catalyzed processes, and the research mainly focused on inorganic acid catalysts (Zhou et al., 2021). For example, in Yemis’ research, inorganic acids such as sulfuric acid, hydrochloric acid, and phosphoric acid were used as catalysts, and the yield of furfural could reach 37.5% (Yemiş and Mazza, 2011). However, there were many disadvantages, such as low yield, equipment corrosion, and post-processing difficulties (Wang et al., 2017). In order to solve the problems of inorganic acids, solid acids began to be proposed as catalysts. The solid acid Nb_2_O_5_-MCM-41 was applied by Garcia-Sancho and the yield of furfural could reach more than 59% (García‐Sancho et al., 2013), but this process was also limited due to poor selectivity and high cost. Therefore, finding a more environmentally friendly and efficient catalyst for the conversion of xylose to furfural is of great significance to the development of the furfural industry and even bioenergy.

As a compound composed entirely of anions and cations, ionic liquid has the advantages of good solubility and designable structure, which allows it to interact well with cellulose and then promote the conversion of biomass (Usmani et al., 2020). Moreover, to achieve better conversion efficiency, the research trend of ionic liquids is shifting from conventional ionic liquids to functionalized ionic liquids. Generally speaking, the functionalized ionic liquid refers to ionic liquids with functional groups, such as acidic groups, basic groups, hydrophilic groups, and hydrophobic groups; then, they will have specific functions. For cations, the ionic liquids used in furfural preparation are mainly of the monoimidazole halide type ([BMIM]Cl (Zhao et al., 2019) and [EMIM]Cl (Yu et al., 2012)). These ILs are usually water-soluble, resulting in the difficulty of separation from the reaction system. Compared with monoimidazole ionic liquids, the diimidazole ionic liquids have a better hydrophobicity at around room temperature and have good thermal stability (Ni et al., 2017; Liu et al., 2019). Obviously, functionalized diimidazole ionic liquids may have great application prospects in the preparation of furfural, but only a few studies were conducted and few applications were discovered.

In this work, in order to achieve a breakthrough in the field of preparation of furfural catalyzed by the diimidazole hexafluorophosphate ILs, a series of diimidazole hexafluorophosphate ILs were synthesized, and then they were applied to explore a feasible way for the synthesis of furfural from xylose under the microwave system. Finally, good experimental results have been obtained.

## Experimental

### Materials and Chemicals

1-Methylimidazole, 1-vinylimidazole, dibromomethane, 1,2-dibromoethane, 1,3-dibromopropane, 1,4-dibromobutane, 1,5-dibromopentane, 1,6-dibromohexane, 1-bromobutane, camphorsulfonic acid (marked as CSA), and potassium hexafluorophosphate were purchased from Aladdin Biochemical Technology Co., Ltd. (Shanghai China); all other reagents were commercially available and were used as received.

### Preparation of Diimidazole Hexafluorophosphate

In this work, the ionic liquids, such as [C_1_MIM]PF_6,_ [C_2_MIM]PF_6_, [C_3_MIM]PF_6_, [C_4_MIM]PF_6_, [C_5_MIM]PF_6_, [C_6_MIM]PF_6_, [C_1_VIM]PF_6,_ [C_3_VIM]PF_6_, and [BMIM]PF_6_, have been synthesized according to the literature (Liu et al., 2011; Zhang et al., 2012; Al-Harthi et al., 2021), and they were marked as C_1_MF, C_2_MF, C_3_MF, C_4_MF, C_5_MF, C_6_MF, C_1_VF, C_3_VF, and BIF, respectively. However, the synthesis of diimidazole hexafluorophosphate [3,3′-methylenebis (1-methyl-1H-imidazol-3-ium), C_1_MF] was somewhat special, and the detailed synthesis process was as follows.

The C_1_MF was synthesized by a two-step method. First of all, an appropriate amount of dibromomethane was added dropwise to the 1-methylimidazole (8.21 g, 0.1000 mol) under stirring at 110°C for 2 h. Subsequently, white solid would be obtained after the reaction was completed and cooled down to room temperature, and the product was washed with ethyl acetate and diethyl ether 3 times, respectively. Afterward, the residual solvent was removed under a rotary evaporator and dried *in vacuo* at 50°C for 30 min. The white powder solid intermediate (C_1_MB) was obtained with a yield of 94.67%.

**FIGURE 1:**
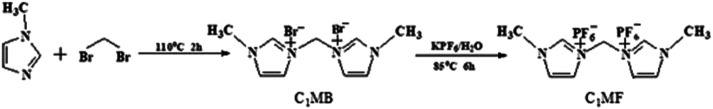
Schematic diagram of the synthesis of C_1_MF.

The second step was focused on the anion exchange reaction. Briefly, the C_1_MB (5.13 g, 0.015 mol) and potassium hexafluorophosphate (5.50 g, 0.030 mol) were dissolved in 50 ml of deionized water. Subsequently, the mixture was stirred at 85°C for 6 h; then, a colorless crystal C_1_MF was obtained with a yield of 78.6%. The scheme is shown in Figure 1.

### Synthesis of Furfural From Xylose

0.3 g (0.002 mol) D (+)-xylose and 5 ml solvent were added to a microwave reaction flask equipped with a stir bar. After D (+)-xylose was dissolved, an appropriate amount of hexafluorophosphate (or another catalyst) was added. Then, the microwave synthesizer (Discover, United States) is run and the reaction proceeded at 205°C for 8 min. After the reaction was completed, the reaction liquid was filtered. Subsequently, a pipette was used to accurately deliver a known volume of solution into a 10 ml volumetric flask, which was diluted it to the specified range (within the furfural standard curve range) for measurement. Compared with the microwave method, the reaction in hydrothermal synthesis reactor was carried out at 150°C for 5.5 h.

### Analyses

The content of furfural was analyzed by HPLC (Waters 2,695, United States) equipped with a C18 reverse chromatographic column and a photo-diode array detector (PDAD). The detection wavelength was 275 nm, and the column temperature was maintained at 313 K. The mixture of acetonitrile and water (3:97, v/v) was used as the mobile phase and at a flow rate of 1 ml/min. At the same time, the detection of furfural yield was carried out with an external standard method. Furfural standard solutions were prepared in deionized water to concentrations of 0.1, 0.2, 0.3, 0.4, and 0.5 mg/ml, and according to the above analysis method for detection. Then, the concentration (mg/ml) was taken as the abscissa and the peak area (µv*s) as the ordinate (Supplementary Figure S1), and the linear regression equation of the standard curve was *y* = 39,903,400 *x* + 406,859.8, correlation: R_2_ = 0.99959.

$$C_{1}=X=\frac{\left(y-406859.8\right)}{39903400},$$

$$C_{2}=\frac{\left(C_{1}\times V_{1}\right)}{V_{2}},$$

$$\mathrm{Yield}\;\mathrm{of}\;\mathrm{furfural}\left(\%\right)=\frac{\left(C_{2}\times V_{3}\right)/M_{furfural}}{mole\;of\;initial\;xylose}\times 100\%,$$

where C_1_ is the concentration measured in HPLC after dilution; C_2_ is the initial concentration of reaction solution; V_1_ is the volume of reaction solution after dilution; V_2_ is the volume of reaction solution used to dilution; V_3_ is the initial volume of the reaction solution.

## Results and Discussion

### Characterization of C_1_MB and C_1_MF

**FIGURE 2:**
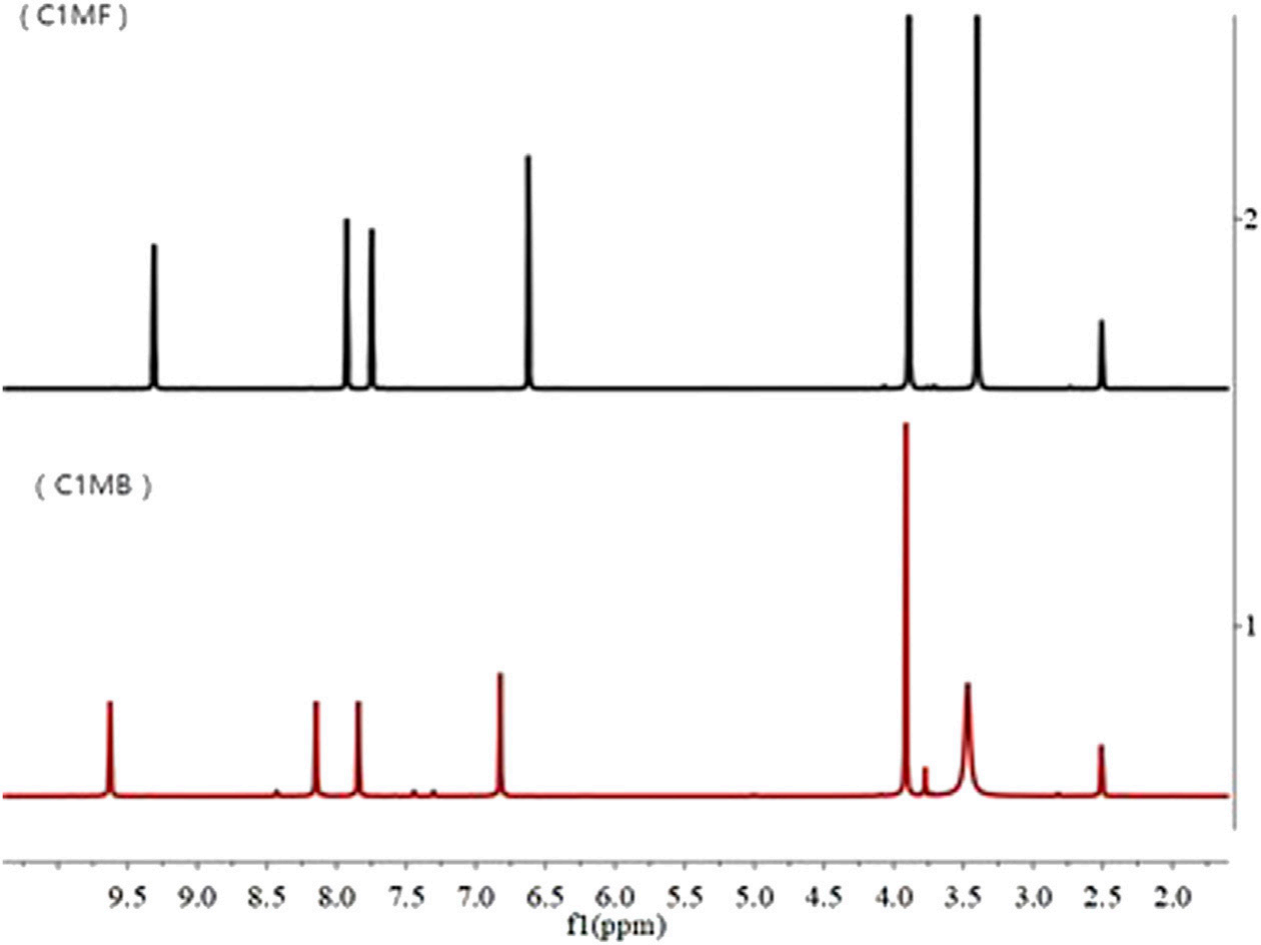
^1^HNMR of the C_1_MB and C_1_MF.

As shown in Figure 2, except for the solvent peaks (2.5 and 3.4 ppm), five different types of protons could be observed in both the C_1_MF and C_1_MB, and the detailed spectrum information was as follows:

C_1_MB: ^1^H NMR (400 MHz, DMSO) δ_ppm_: 9.63 (s, 1H), 8.15 (d, J = 1.8 Hz, 1H), 7.84 (d, J = 1.7 Hz, 1H), 6.82 (s, 1H), 3.91 (s, 3H).

C_1_MF: ^1^H NMR (400 MHz, DMSO) δ_ppm_: 9.31 (s, 1H), 7.93 (d, J = 1.8 Hz, 1H), 7.75 (d, J = 1.7 Hz, 1H), 6.62 (s, 1H), 3.89 (s, 3H).

According to the structures and properties of target ILs, the chemical shifts of 7.75 and 7.93 ppm and 9.31 ppm belong to imidazole protons of C_1_MF, while the methyl proton had a chemical shift of 3.89 ppm and the methylene proton had a chemical shift of 6.62 ppm. Accordingly, the chemical shifts 7.84 and 8.15 ppm and 9.63 ppm belong to the imidazole protons of C_1_MB, and the methyl and the methylene proton had chemical shifts of 3.91 and 6.82 ppm, respectively. In addition, the phenomenon that the chemical shift was deshielded by the positively charged imidazole ring could be observed when the anion Br^−^ was replaced with PF_6_^−^.

**FIGURE 3:**
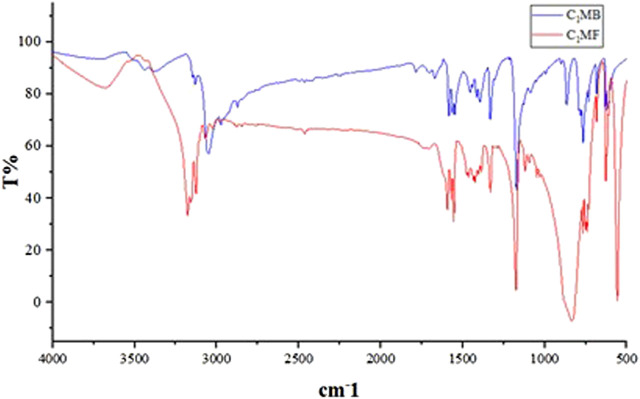
IR of the C_1_MB and C_1_MF.

As shown in Figure 3, the absorption peaks of the sample at 3,000–3,100 cm^−1^ and 1,200 cm^−1^ were attributed to the = C-H of the imidazole rings stretching vibrations. The C=N- on the imidazole-based stretching vibrations, -CH_2_- stretching vibrations, and the related in-plane -CH_2_- bending appearing at 1,500 cm^−1^, 2,900 cm^−1^, and 1,450 cm^−1^ were also observed, respectively. From the fact that the wavenumber of -CH_2_- was larger than the normal value, it could be seen that the positive charge effect of the imidazole ring was obvious, confirmed by the chemical shift of the methylene group (6.82 ppm). Moreover, the chemical shift of the methyl proton was approximately 3.90 ppm, which further proved the existence of C=N-. In addition, the characteristic absorption of hexafluorophosphate was the strong absorption peaks near 830 and 558 cm^−1^.

### Preliminary Exploration of the Reaction

In order to explore the influence of the heating methods on furfural yield, the reaction was carried out in both the hydrothermal synthesis reactor and microwave. The experimental results are shown in Table 1.

**TABLE 1:** The effect of heating method and solvent on reaction.

Entry	Heating method	Solvent	Imidazole crystals	Time	Temperature (°C)	Yield %
1	Reactor	EtOH	1 g C_1_MF	5.5 h	150	3.50
2	Reactor	DMSO	1 g C_1_MF	5.5 h	150	1.17
3	Reactor	H_2_O	1 g C_1_MF	5.5 h	150	25.68
4	Reactor	H_2_O	1 g C_1_VF	5.5 h	150	20.27
5	Reactor	H_2_O	1 g C_3_VF	5.5 h	150	19.34
6	Reactor	H_2_O	1 g BIF	5.5 h	150	15.88
7	Microwave	EtOH	1 g C_1_MF	10 min	175	2.24
8	Microwave	DMSO	1 g C_1_MF	10 min	200	20.86
9	Microwave	H_2_O	1 g C_1_MF	10 min	200	40.70
10	Microwave	H_2_O	1 g C_1_VF	10 min	200	34.87
11	Microwave	H_2_O	1 g C_3_VF	10 min	200	39.14
12	Microwave	H_2_O	1 g BIF	10 min	200	32.27

Reaction conditions: xylose (60 g/L).

It could be seen from Table 1 that the different heating methods had different degrees of influence on the yield of furfural. As a closed system, the hydrothermal synthesis reactor was conducive to the steady increase of the reaction temperature. In addition, the increase of the reaction temperature was beneficial to the reaction since the synthesis of furfural by dehydration of xylose is an endothermic reaction. However, the yield of furfural was lower in the hydrothermal reactor, which might be due to the inability to stir during the reaction, so further affecting the mass transfer and heat transfer. In recent years, microwave technology had made many major achievements in the field of catalytic biomass conversion (Moodley and Kana, 2017; Teh et al., 2017). Some researchers have used microwaves to assist the conversion of fructose into 5-hydroxymethyl furfural, which has also achieved good results (Zhu et al., 2016; Shao et al., 2021). Therefore, the microwave method has also been introduced into the preparation of furfural in this work. Under the microwave method, the reaction time for the conversion of xylose to furfural can be effectively shortened from 5.5 h to 10 min, and the furfural yield could reach 40.70%. For the microwave method, the shorter reaction time would greatly reduce energy consumption, which also laid a good foundation for its development.

The research results also showed that the catalytic activity of C_n_MF was higher than that of C_n_VF, which might be due to the stronger electron donating ability of methyl group than that of vinyl group so it could promote the formation of the positive charge of imidazole ring and subsequent proton transfer. Therefore, the catalytic activity of ILs will change to a certain extent with the structure of the substituted group (R-) on the imidazole ring changing. On the other hand, the experimental results showed that the catalytic activity of the diimidazole hexafluorophosphate catalyst was better than that of monoimidazole. In order to have a better understanding of these questions, the structure of the ionic liquids had been given in Supplementary Figure S2.

According to Table 1, the different solvents also influenced the furfural yield. As we all know, DMSO was usually used as the reaction solvent in the traditional preparation process of furfural or 5-hydroxymethyl furfural for its excellent dissolubility. However, using DMSO as a solvent in this work, the furfural yield was only 20.86% under the microwave and even only 1.17% when the reaction was carried out in the hydrothermal synthesis reactor. Analyzing the situation in combination with experimental phenomena, it can be found that at high temperatures, DMSO will partly undergo disproportionation reaction and the other part will undergo decomposition reaction (Nishimura et al., 1972). Moreover, it will decompose itself into methyl mercaptan and further transform into methyl sulfide when reaction temperature exceeds 130°C (Lian et al., 2015). When EtOH was used as the reaction solvent, the furfural yield was not higher than 3.50%. However, when H_2_O was used as a solvent, the furfural yield could reach 40.70% under the same conditions. Considering the advantages of green, environmental protection and being cheap and easy to obtain, water will be used as a solvent in the experiments to explore the preparation of furfural from xylose in the following section.

### Preparation of Furfural *via* Diimidazole Hexafluorophosphate Catalyzed

**FIGURE 4:**
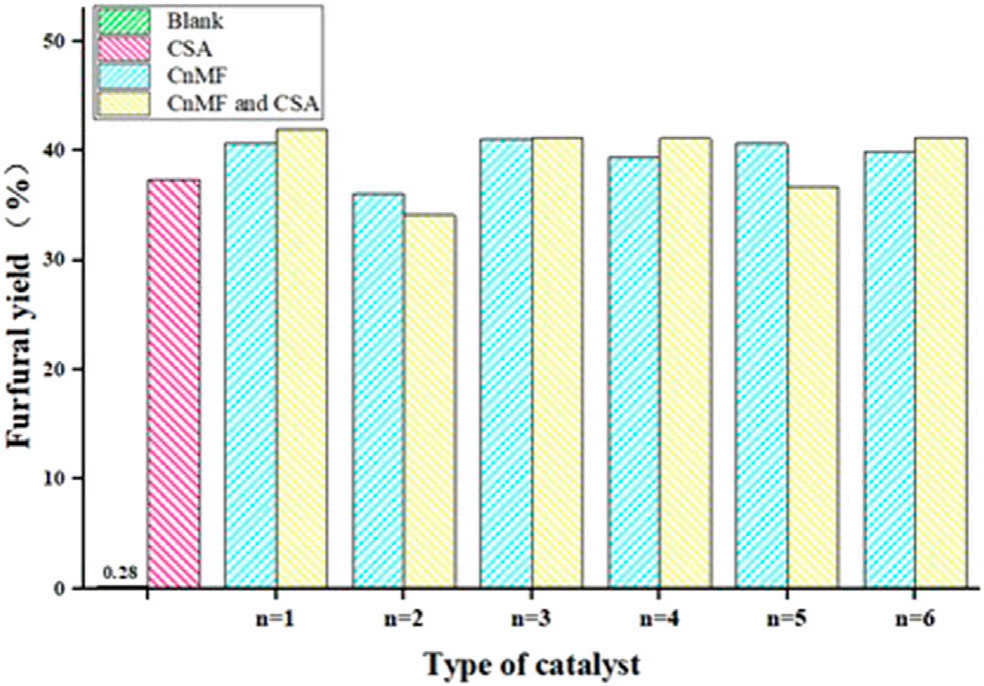
The effect of catalyst type on reaction. Reaction conditions: xylose (60 g/L), 0.1 g CSA, 1 g C_n_MF, 200°C, 10 min.

To investigate the catalytic activity of diimidazole hexafluorophosphate, six diimidazole hexafluorophosphates (C_1-6_MF) with different carbon chain lengths were applied to the preparation of furfural from xylose by dehydration; the experimental results are shown in Figure 4.

As we can see, different catalyst types had different degrees of influence on the yield of furfural. When six C_n_MF were used as catalysts, respectively, the furfural yield was approximately 36.10–40.99%, and C_3_MF gave the highest yield of 40.99%. However, the change in the length of the carbon chain between the imidazole rings only had a slight influence on the furfural yield. It was worth mentioning that the yield of furfural was only 0.28% in the case of the IL blank, which meant that the catalytic activity of C_n_MF exceeds 35%, and there was an obvious improvement compared with that in the conventional heating method. Considering that the acidity was necessary for converting xylose into furfural, a co-catalyst was further added to the reaction.

Camphorsulfonic acid, as a novel solid acidic catalyst, could be prepared by a one-step reaction of camphor powder and concentrated sulfuric acid under mild conditions, and the yield could reach 85%, which also laid a good foundation for its application (Liu et al., 2013; Kaur et al., 2018). Therefore, in this work, a small amount of camphorsulfonic acid as a co-catalyst was introduced into the reaction. However, the result did not seem to be more effective than that of the single one, and even a partial decline can be observed. At the same time, there was an interesting phenomenon: the better the crystal quality, the fewer the by-products in the system after the reaction. Therefore, it was preliminarily inferred that the yield of furfural might be affected by the crystal quality, but there was a need for more in-depth research.

### Experimental Results of Optimized Reaction Conditions

**FIGURE 5:**
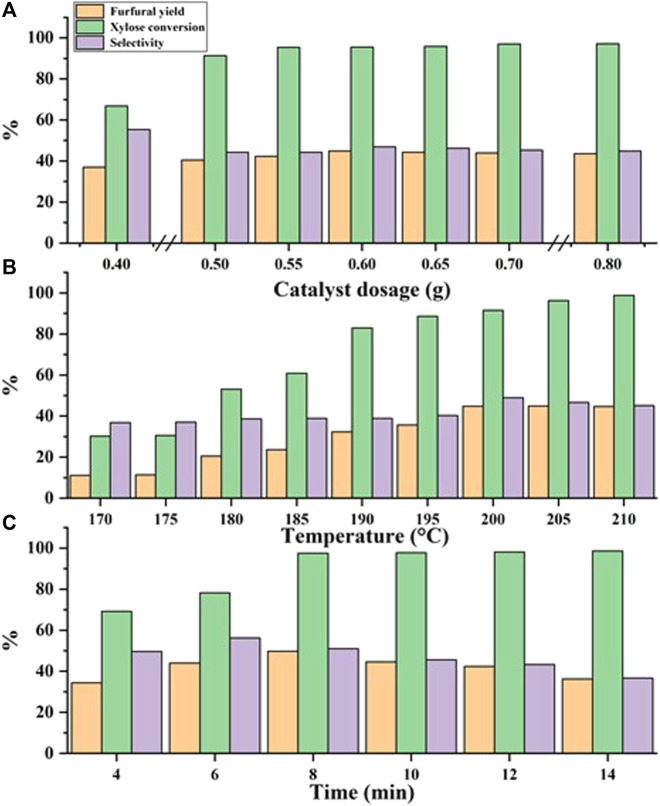
Experimental results of optimized reaction conditions. Reaction conditions: **(A)** xylose (60 g/L), 200°C, 10 min; **(B)** xylose (60 g/L), 0.6 g C_1_MF, 10 min; **(C)** xylose (60 g/L), 0.6 g C_1_MF, 205°C.

Based on the above, the effect of different reaction conditions on the dehydration of xylose to furfural was investigated, and the results are given in Figure 5.

Figure 5A exhibited the results of furfural yield was affected by the different catalyst dosages. The reaction proceeded at 200°C for 10 min, and the same initial concentration of xylose was 60 g/L. It followed a law of initial increase and subsequent decrease with the increase of the dosage of C_1_MF. It is speculated that xylose is easily converted into active intermediates (like Humin) in the process of xylose dehydration to produce furfural, and then the active intermediates are further converted into furfural. At the same time, since ionic liquids can well promote the conversion of biomass, the continuous increase in the dosage of C_1_MF was helpful to promote the dissolution of xylose, which was continuously converted into furfural. However, the catalytic activity of C_1_MF will not enhance significantly with increasing the C_1_MF dosage because the C_1_MF has poor solubility in water at low temperatures, and is difficult to dissolve in time. This will greatly increase the possibility of collisions between furfural and xylose or reaction intermediates, leading to side reactions (Zhao et al., 2016).

Given that the reaction temperature is an important factor, which greatly influences the formation of furfural, the effect of different reaction temperature on furfural yield was subsequently tested. It could be seen from Figure 5B that the yield of furfural would be increased in a certain range with the increase of reaction temperature, which indicated that xylose would continue to be converted to furfural as the reaction proceeds. Therefore, when the reaction temperature is close to 205°C, xylose could be converted into furfural to the greatest extent. The reason for this phenomenon might be that the dehydration of xylose to furfural was an endothermic reaction, and a higher temperature was beneficial to the increase of furfural yield. However, when the temperature exceeds 205°C, the furfural yield began to decline, which might be due to the side reaction, such as the condensation reaction between product furfural and intermediate products or the resinification reaction of furfural itself (Sun et al., 2012).

Of course, it could also be found from Figure 5C that the yield of furfural was also significantly affected by the reaction time. As the reaction was prolonged, it was conducive to the full conversion of xylose into furfural in the initial stage of the reaction, resulting in a continuous increase in furfural yield. When the reaction time was prolonged to 8 min, the yield of furfural reached a higher level. However, when the reaction time was prolonged further to 8 min, the furfural yield gradually decreased. The possible reason was that the degradation, resinification, and condensation would take place with the extension of the reaction time (Zhao et al., 2016).

Through continuous in-depth exploration, the optimal yield of 49.76% could be obtained in the aqueous phase at 205°C for 8 min. This optimal yield showed a desirable result compared to the literature reported, in which the reactions were also carried out in the aqueous phase. In the case of the molecular sieve catalyst proposed by Chen, the reaction condition was 2.0 g of molecular sieve catalyst, 50 ml of distilled water, 100 ml of toluene, a reaction time of 4 h, and a reaction temperature of 170°C, and the maximum yield of furfural was only 29.2% (Chen et al., 2007). Likewise, in another related work, 1-ButOH and H_2_O were used as the solvents and the MCM-41 molecular sieve was used as the catalyst, the reaction was carried out at 170°C for 4 h, and the final furfural yield was close to 44.05% (Zhang and Lin, 2010). Herein, compared with some other reports, not only was the yield was higher than some other reports, but also the reaction time was significantly shortened and even the reaction solvent was more green and environmentally friendly. In addition, in recent years, research on the preparation of furfural from xylose catalyzed by solid acid in the water phase under the microwave had also been tried by Gerardo, and the results showed that the furfural yield was 48% (Gómez Millán et al., 2018), which was slightly lower than that of this work.

### Reusability of the Catalyst

**FIGURE 6:**
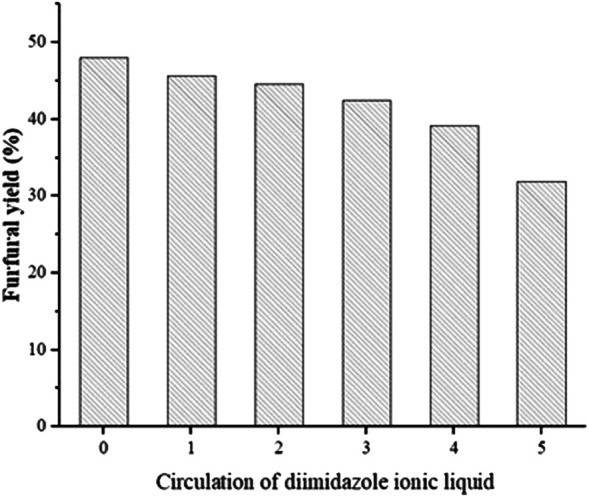
The reusability of the catalyst. Reaction conditions: xylose (60 g/L), 0.6 g C_1_MF, 205°C, 10 min.

The recycling of C_1_MF was tried under the optimal conditions to study the reusability of the catalyst. The procedure of the recycled experiment was as follows: the mixed solution was collected by centrifugation and filtration; then, the furfural was separated from the reaction solution by anhydrous ether extraction. Subsequently, the aqueous phase after extraction was tested by TLC and HPLC to ensure that the product furfural had been removed. After the appeal operation, the rotary evaporator was used to remove water and residual ether to obtain a concentrated catalyst and then was directly reused. The results of the catalyst recycle test are shown in Figure 6. It was clearly shown that a moderate 31.97% furfural yield was provided by the C_1_MF catalyst after five successive runs, which might be because the structure of the catalyst has been destroyed after a series of cycles.

### Possible Mechanism of Conversion of Xylose to Furfural

**FIGURE 7:**
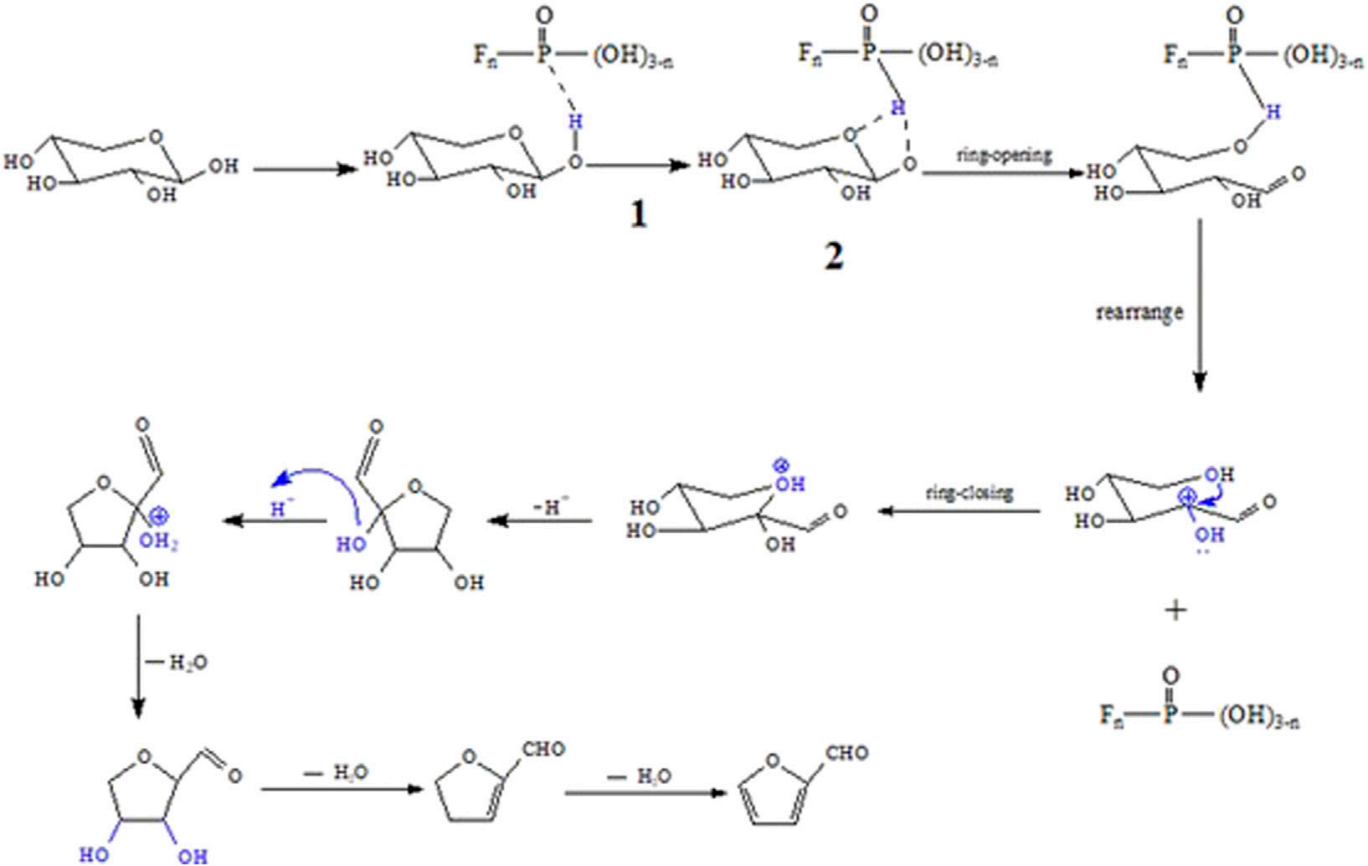
Possible mechanism of the conversion of xylose to furfural catalyzed by C_n_MF in water.

According to the results of the previous section, the furfural yield *via* C_1_MF catalyzed was markedly more than that of the blank experiment (0.28%), and the catalytic activity of diimidazole hexafluorophosphate was better than that of monoimidazole hexafluorophosphate. In order to further explore the possible mechanism, a series of experiments on the different types of catalysts were carried out. First, the C_1_MB was applied in the dehydration of xylose to furfural. The furfural yield was just 13.48%, which was much lower than that of C_1_MF (49.76%), indicating that the anion would affect the reaction markedly. In the subsequent experiment, KPF_6_ was introduced in the reaction, and the result showed that the furfural yield could reach 38.37%, which further proved that the anion (PF_6_^−^) plays a main role in the reaction. At the same time, the cationic imidazole ring also had a certain promoting effect on the reaction, which could also be consistent with the better catalytic activity of diimidazole hexafluorophosphate than that of monoimidazole. It was worth emphasizing that a strong acidity was observed in the final reaction system when the anion of the catalyst was PF_6_^−^. As we know, it was obviously inconsistent with the acidity of hexafluorophosphate (neutral compound). It was preliminarily speculated that this strong acidic environment was provided by the hydrolysis of PF_6_^−^ to HPO_2_F_2_, H_2_PO_3_F, H_3_PO_4_, and so forth. The hydrolysis of hexafluorophosphate has been studied in detail by Terborg et al. (2012), and they also proposed that the solution would be acidic after hexafluorophosphate hydrolysis. According to the references, if the PF_6_^−^ was hydrolyzed to produce monofluorophosphoric acid or difluorophosphoric acid, the pH of the corresponding solution could reach 0.42 and 0.29. On the other hand, if the PF_6_^−^ was completely hydrolyzed to produce phosphoric acid, the pH value would be 1.50. However, whether C_n_MF or KPF_6_ was used, the pH of the final reaction solution was about 1.1 in this work, belonging to the range of 0.29–1.50. In the follow-up case, the equivalent H_3_PO_4_ was applied in the reaction, and the results showed that the yield of furfural was only 25%, which was lower than that of C_1_MF. So, there was reason to believe that the hexafluorophosphate was not completely hydrolyzed, and the intermediate (POF_n_ (OH)_3-n_) of the hydrolysis product was the key compound promoting proton transfer and subsequent dehydration reactions during the reaction. In addition, we believe that the mutual attraction between anions and imidazole ring in the ionic liquid will form a strong electrostatic field to promote the dissolution of xylose, which has been confirmed by Mr. Hu several years ago (Hu et al., 2013). Furthermore, the transfer of proton in the first step is the most crucial to the reaction, which provides an acidic environment for the compound. The possible mechanism of this process is shown in Figure 7.

## Conclusion

In this work, diimidazole hexafluorophosphate, particularly the C_1_MF, was applied in the conversion of xylose into furfural in an aqueous phase under the microwave method, and the experimental results are summarized as follows:

1) Diimidazole hexafluorophosphate ionic liquids can catalyze the conversion of xylose to furfural, and the furfural yield was approximately 36.10–40.99%. Meanwhile, the catalytic activity of C_n_MF was higher than that of C_n_VF, and the catalytic activity of diimidazole hexafluorophosphate catalyst was better than that of monoimidazole hexafluorophosphate.
2) Through a series of optimizations of the reaction, the yield of 49.76% was obtained at 205°C for 8 min. Moreover, the microwave method greatly shortens the reaction time, which was only 1/40 of the time required by the conventional method.
3) By concentrating and recovering the catalyst, its catalytic activity was still available after being recycled five times under optimal conditions, which not only realizes the recycling of the catalyst but also conforms to the development of today’s green chemistry.

This experiment provided a novel way to prepare furfural in the water phase by microwave assistance, which has the characteristics of short time and low energy consumption. In addition, this may provide a certain reference and basis for the conversion of other sugars.

## Data Availability

The original contributions presented in the study are included in the article/Supplementary Material; further inquiries can be directed to the corresponding author.
