# Supplementary material for: Preparation of Furfural From Xylose Catalyzed by Diimidazole Hexafluorophosphate in Microwave
Source: Front Chem. 2021 Sep 1;9:727382. doi: 10.3389/fchem.2021.727382 (PMC8440960; doi:10.3389/fchem.2021.727382)
Supplement: Supplementary file 1 [file datasheet1.pdf]

Figure 1: Standard curve diagram of furfural

Figure 2: The structure of the ionic liquids

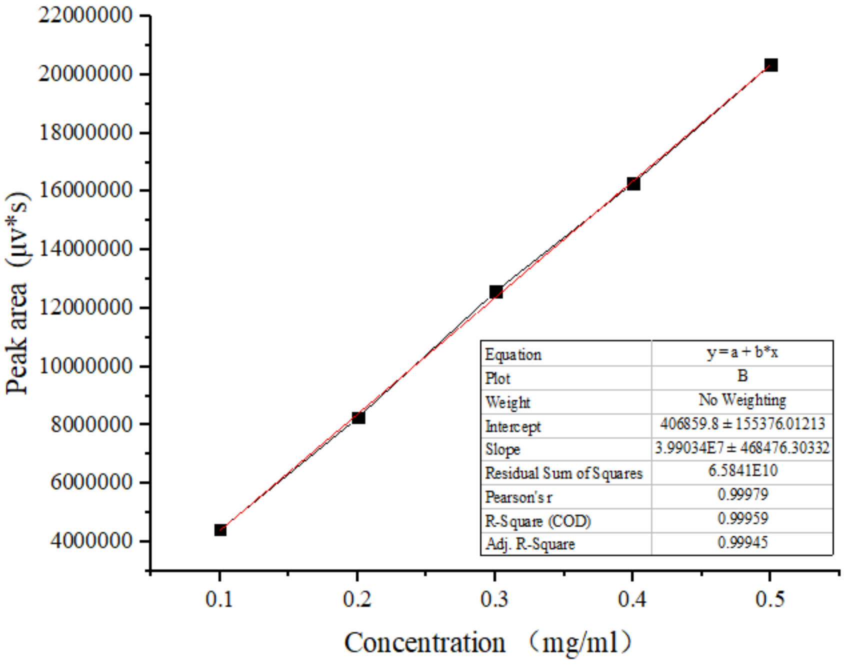

Figure 1: Standard curve diagram of furfural

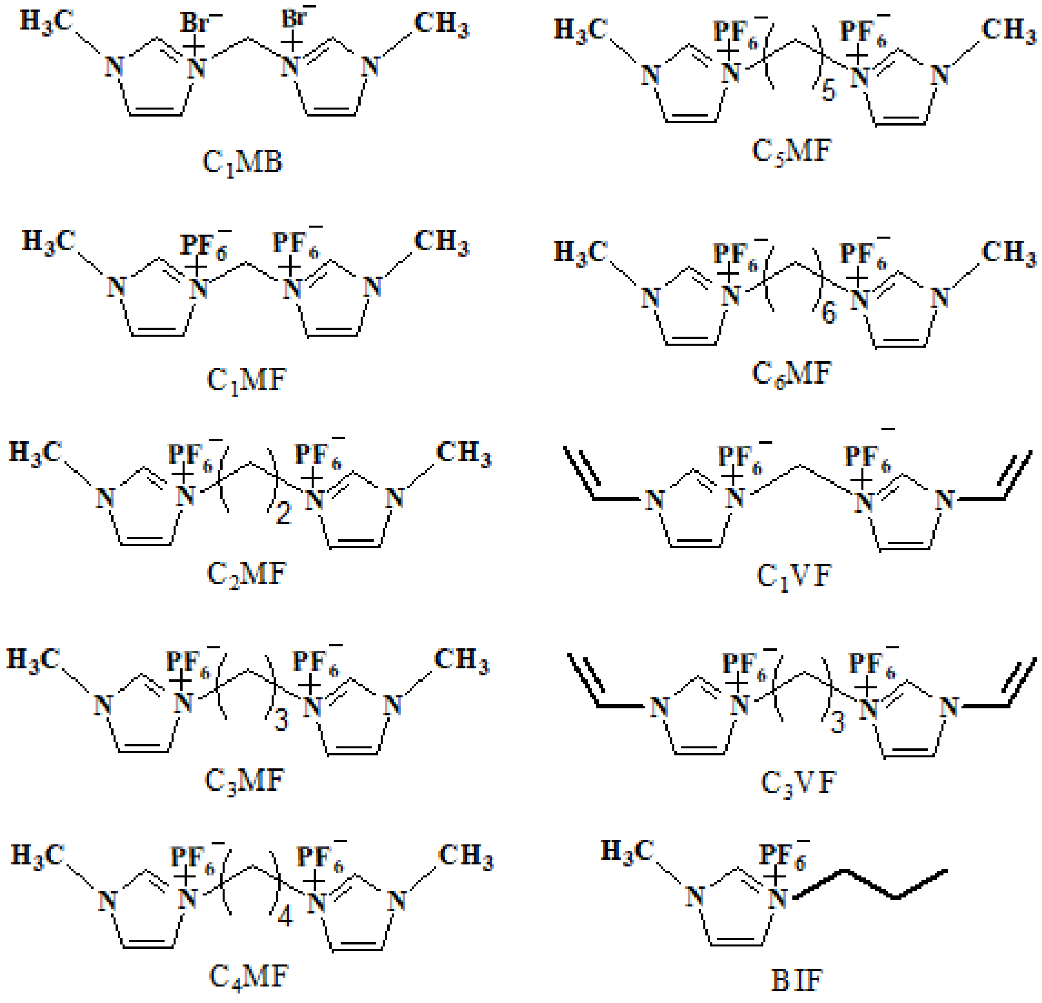

Figure 2: The structure of the ionic liquids
